# Supplementary figures and images for: First photographic evidence of oceanic manta rays (Mobula birostris) at two locations in the Fiji islands
Source: PeerJ. 2022 Sep 7;10:e13883. doi: 10.7717/peerj.13883 (PMC9463998; doi:10.7717/peerj.13883)

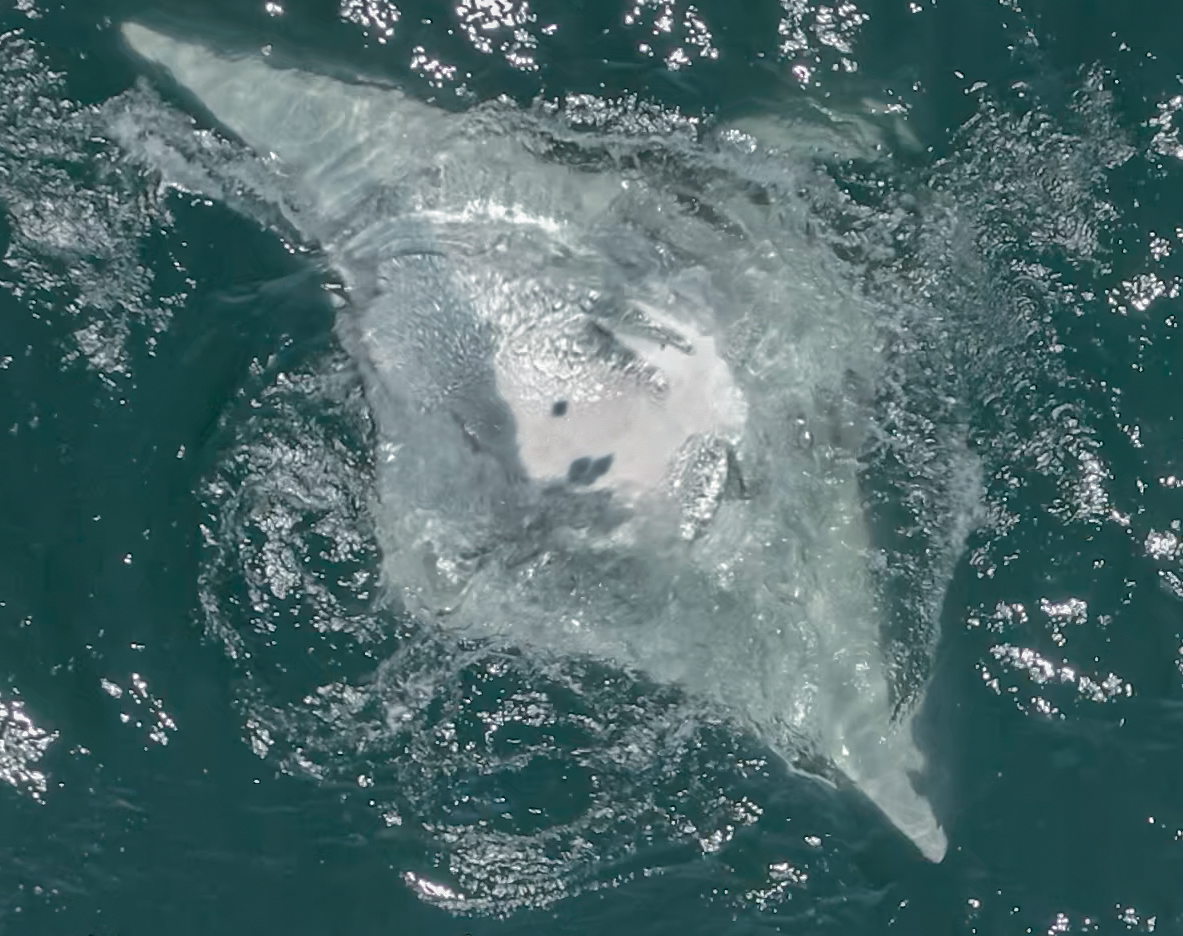

Supplement: Supplemental Information 2 [file peerj-10-13883-s002.jpg]

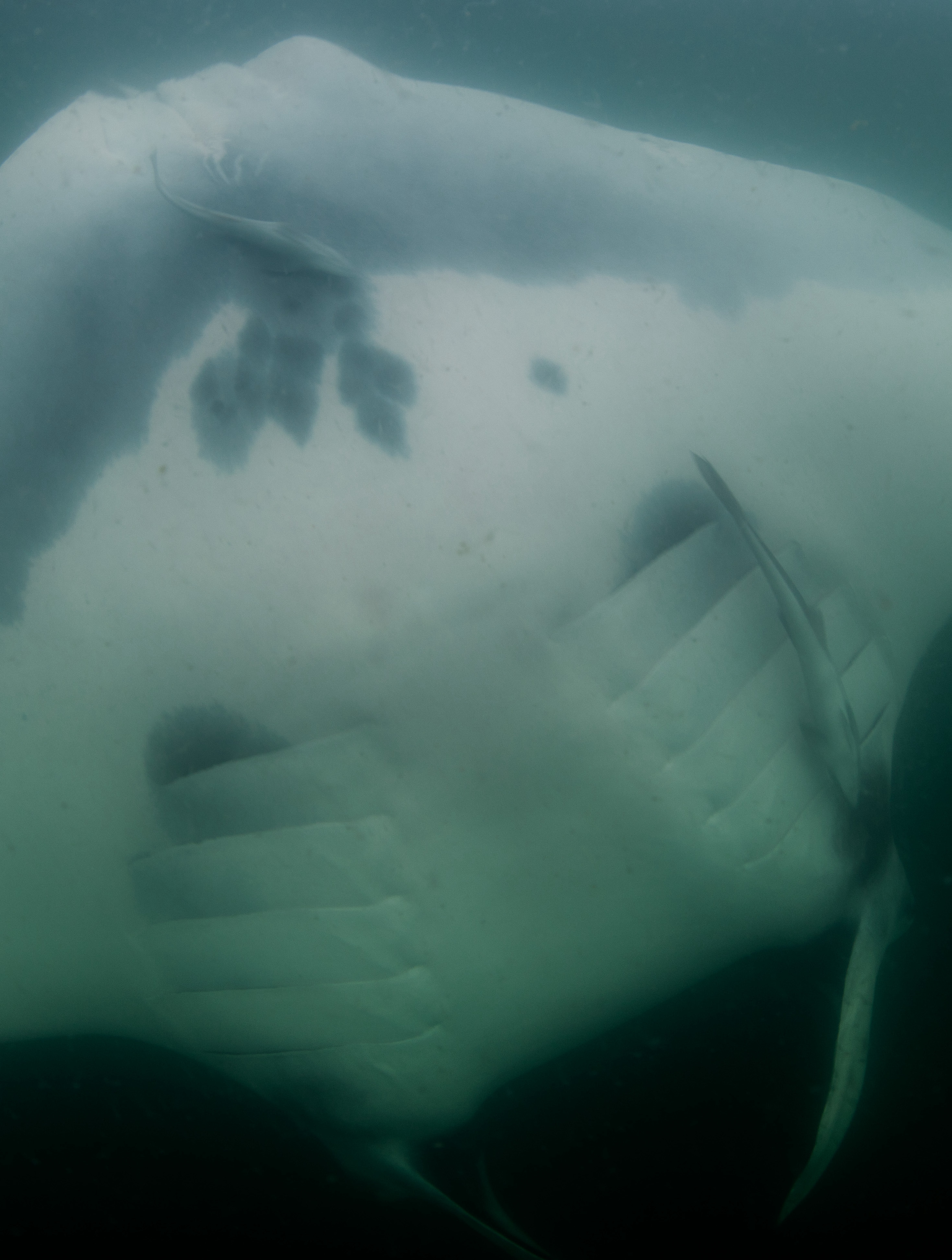

Supplement: Supplemental Information 3 [file peerj-10-13883-s003.jpg]

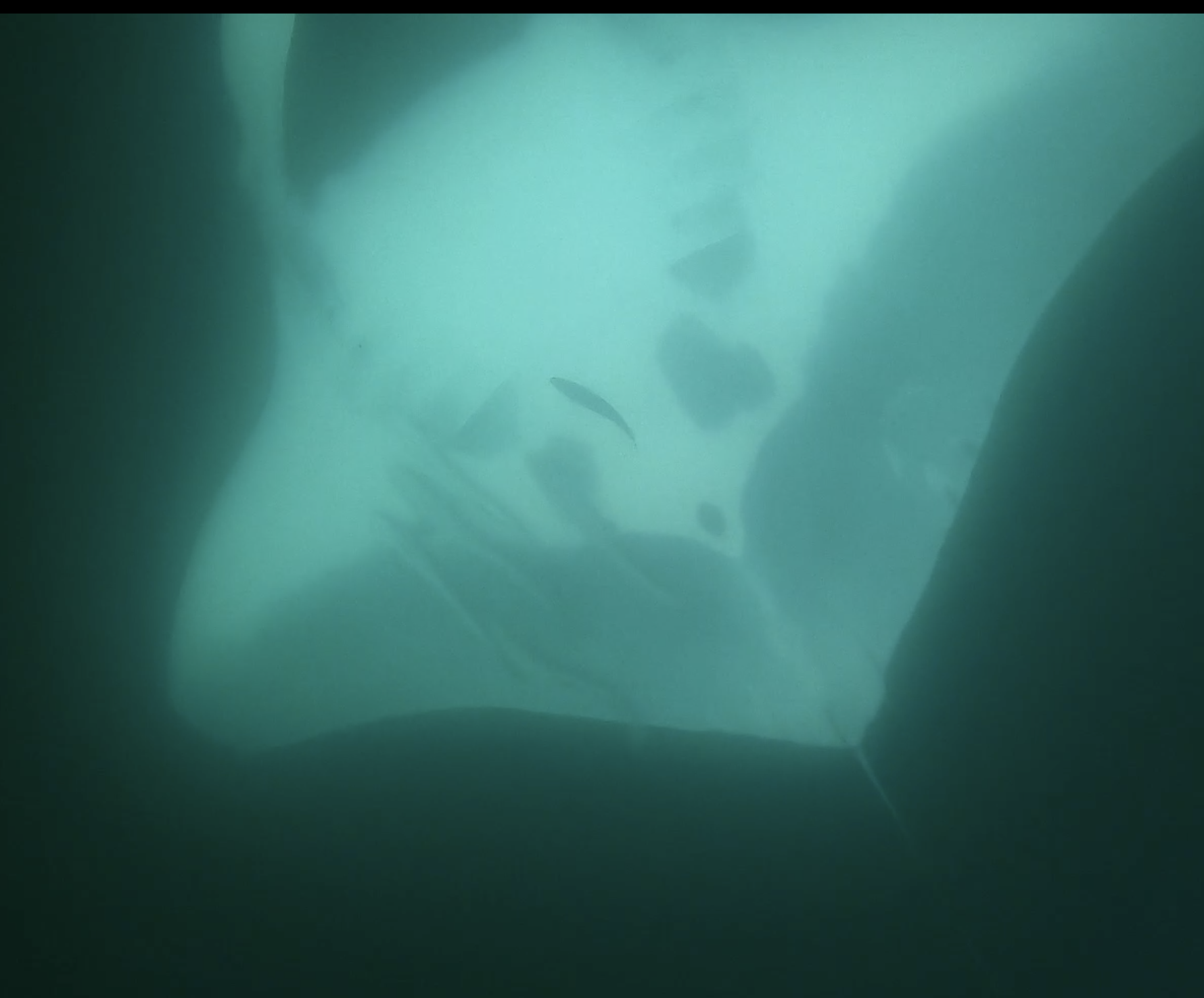

Supplement: Supplemental Information 4 [file peerj-10-13883-s004.png]

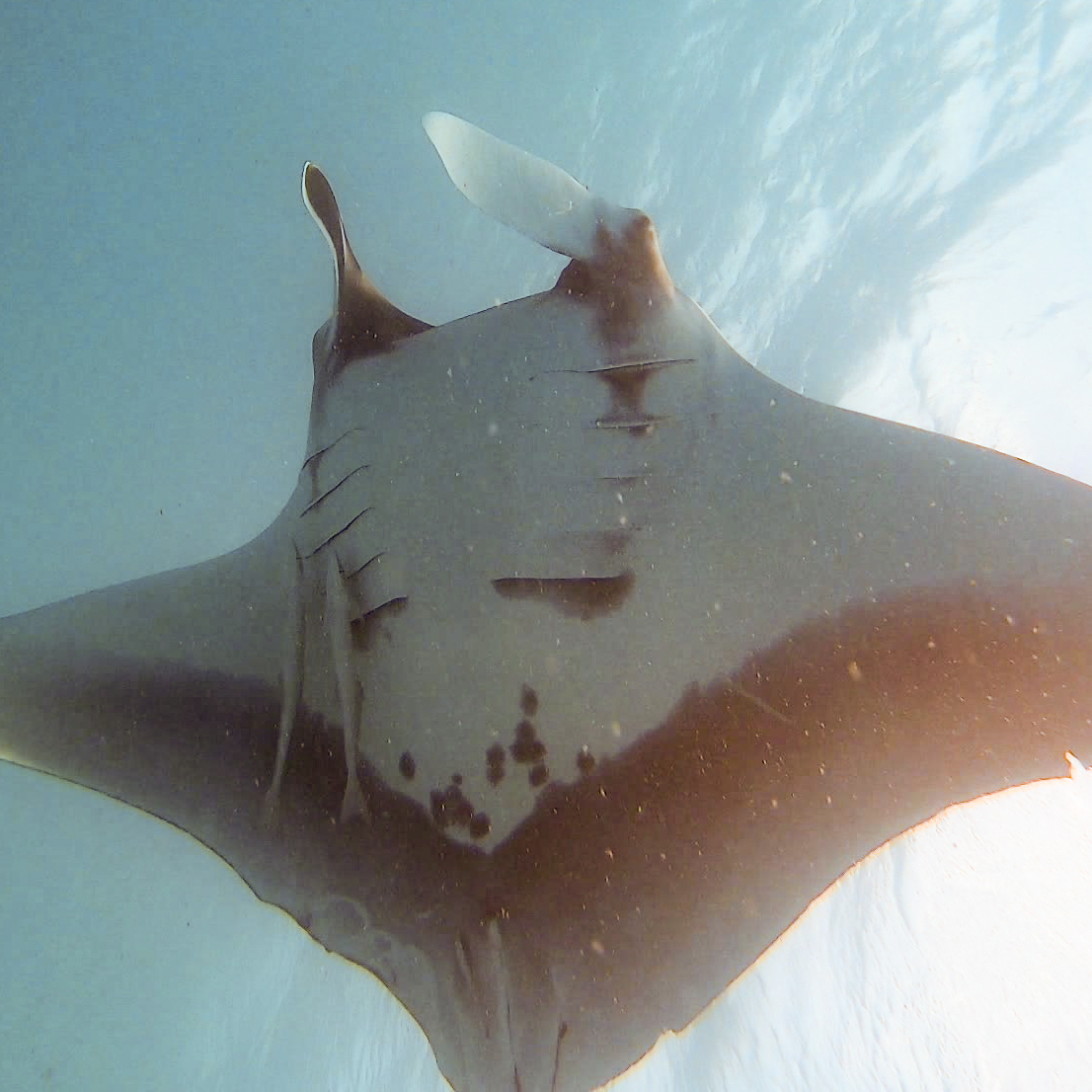

Supplement: Supplemental Information 5 [file peerj-10-13883-s005.jpg]

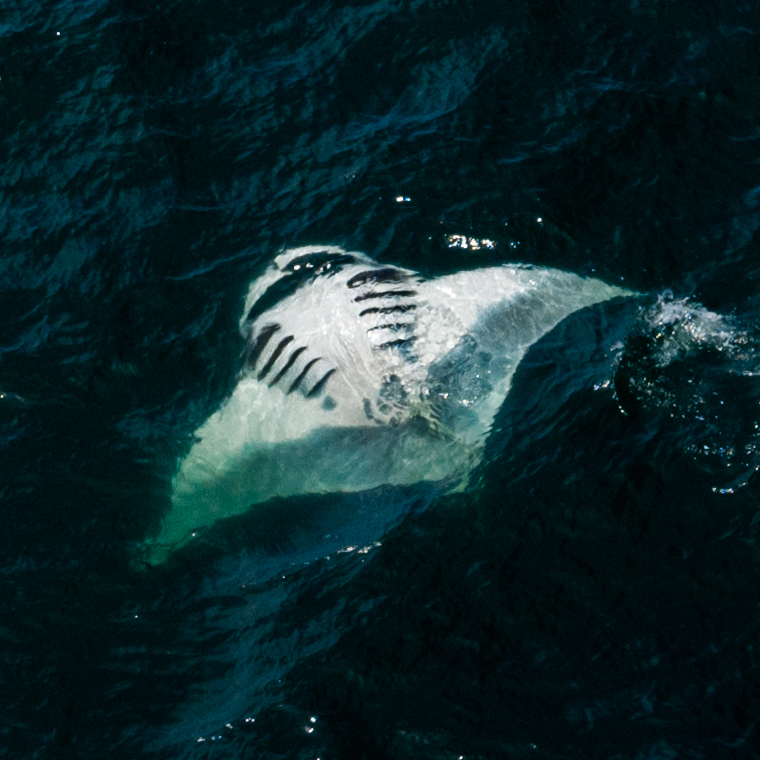

Supplement: Supplemental Information 6 [file peerj-10-13883-s006.jpg]

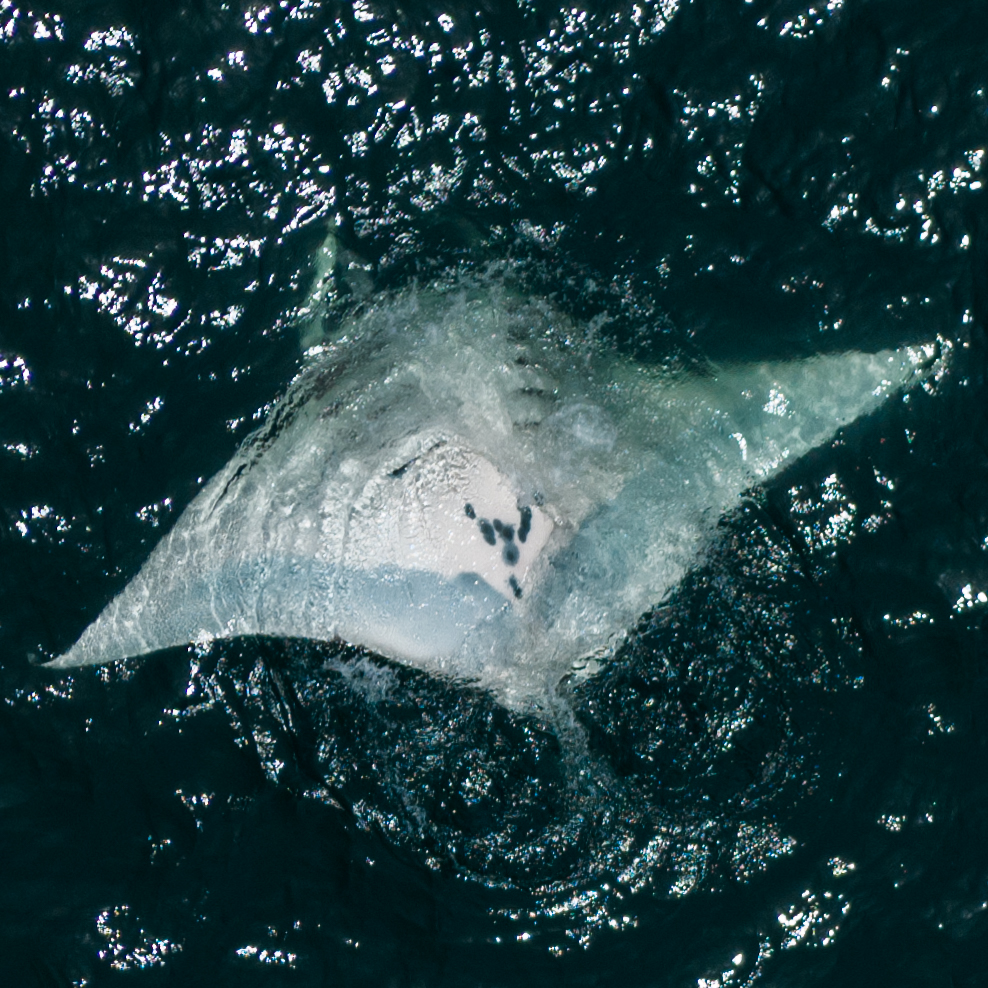

Supplement: Supplemental Information 7 [file peerj-10-13883-s007.jpg]

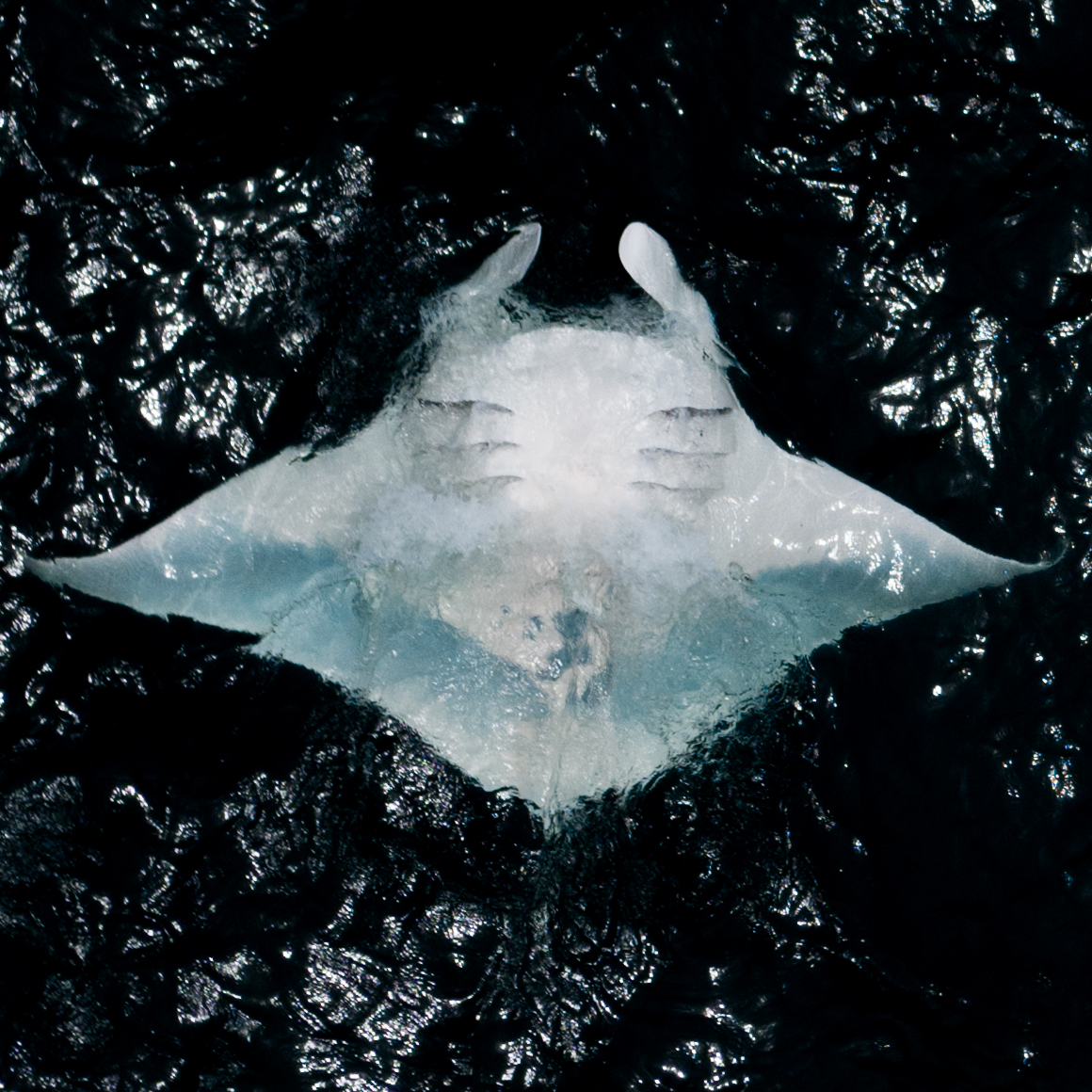

Supplement: Supplemental Information 8 [file peerj-10-13883-s008.jpg]

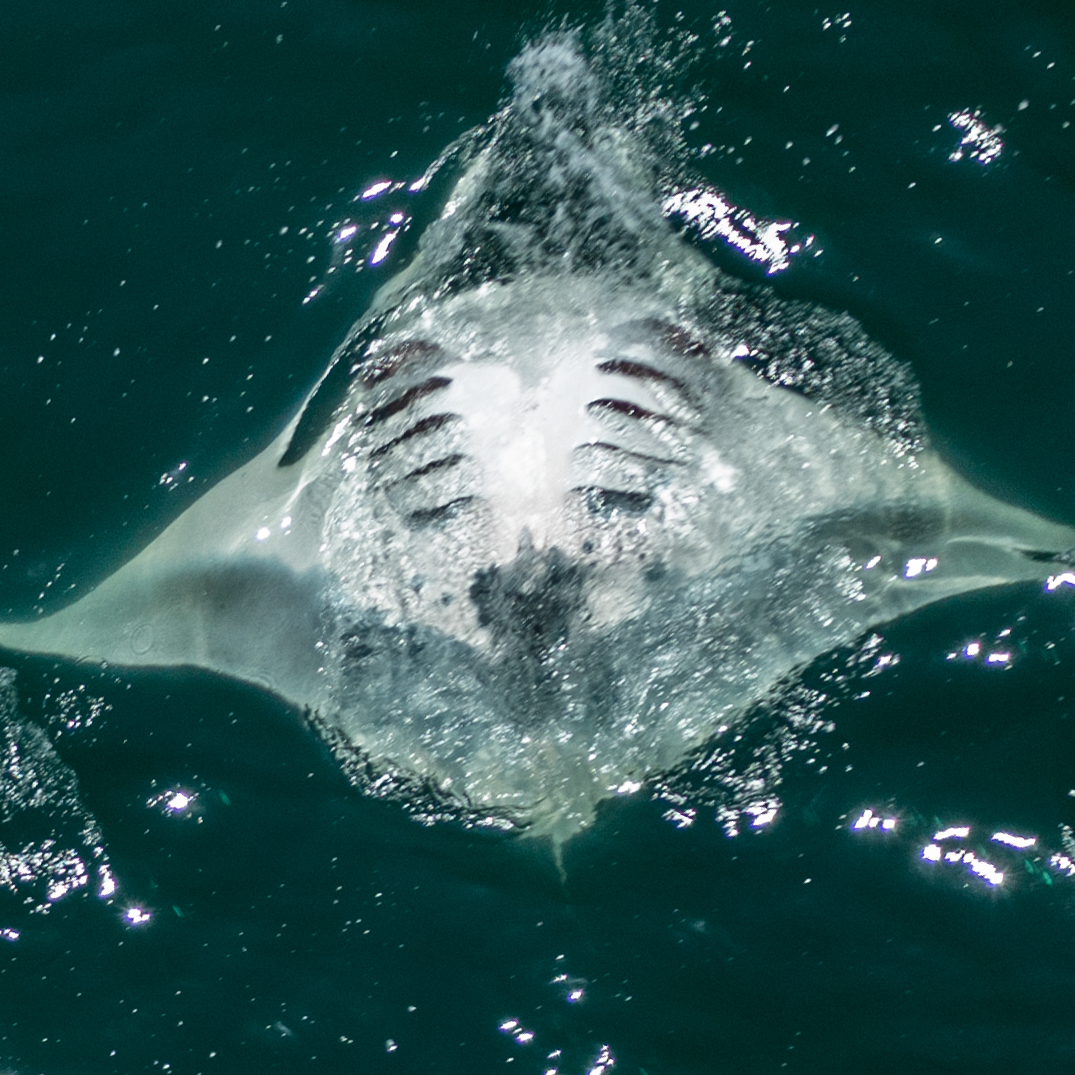

Supplement: Supplemental Information 9 [file peerj-10-13883-s009.jpg]

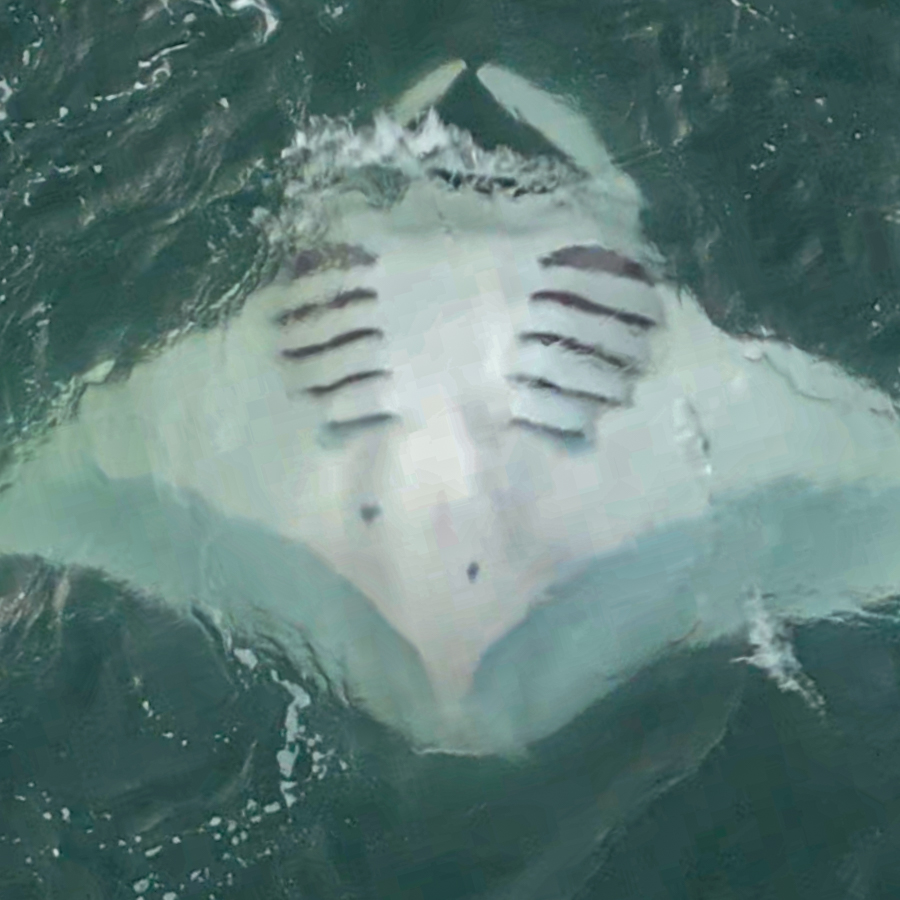

Supplement: Supplemental Information 10 [file peerj-10-13883-s010.jpg]

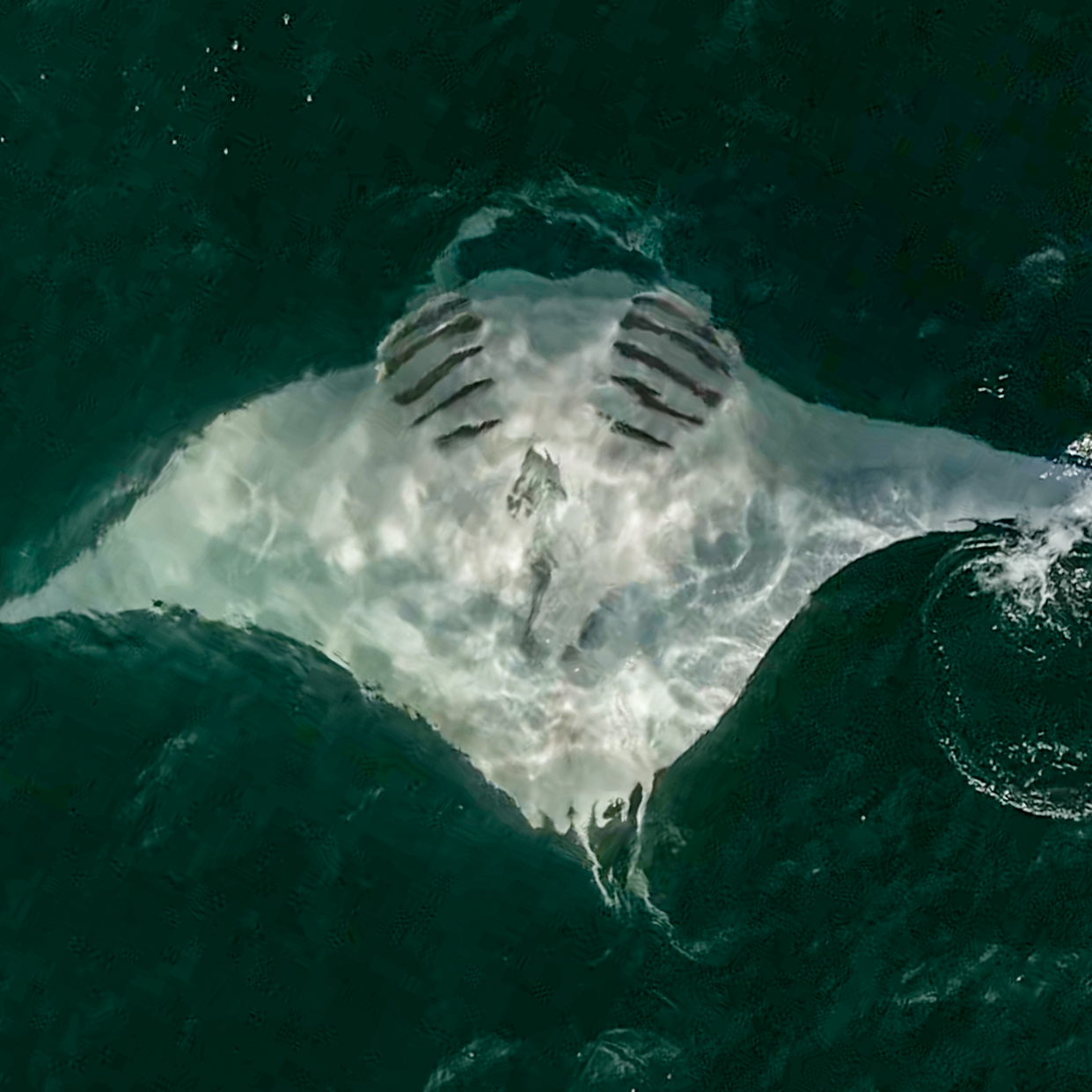

Supplement: Supplemental Information 11 [file peerj-10-13883-s011.jpg]

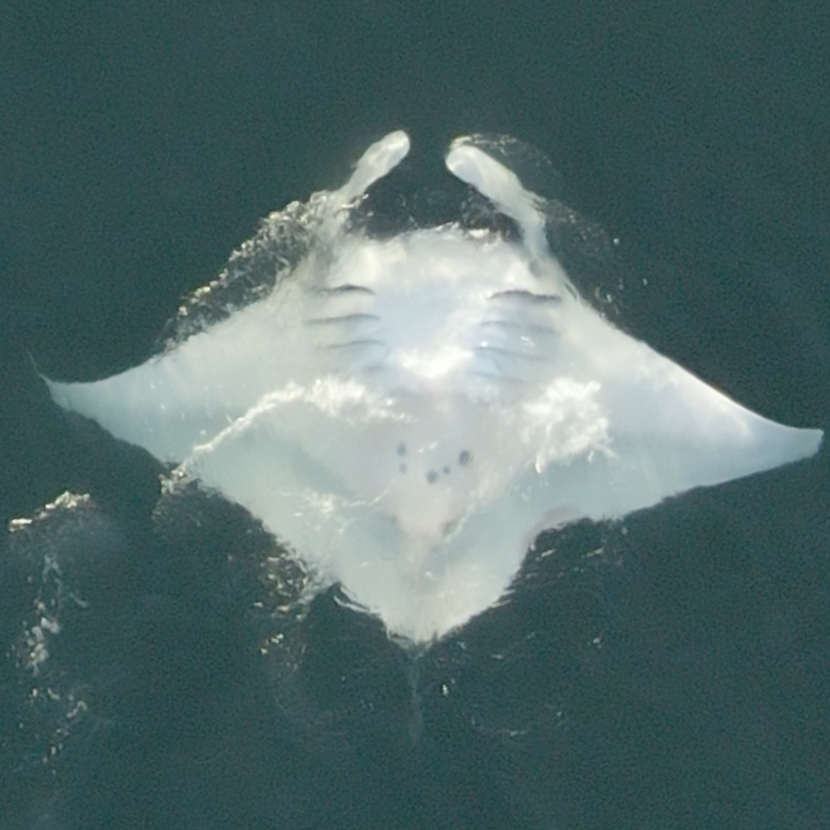

Supplement: Supplemental Information 12 [file peerj-10-13883-s012.jpg]

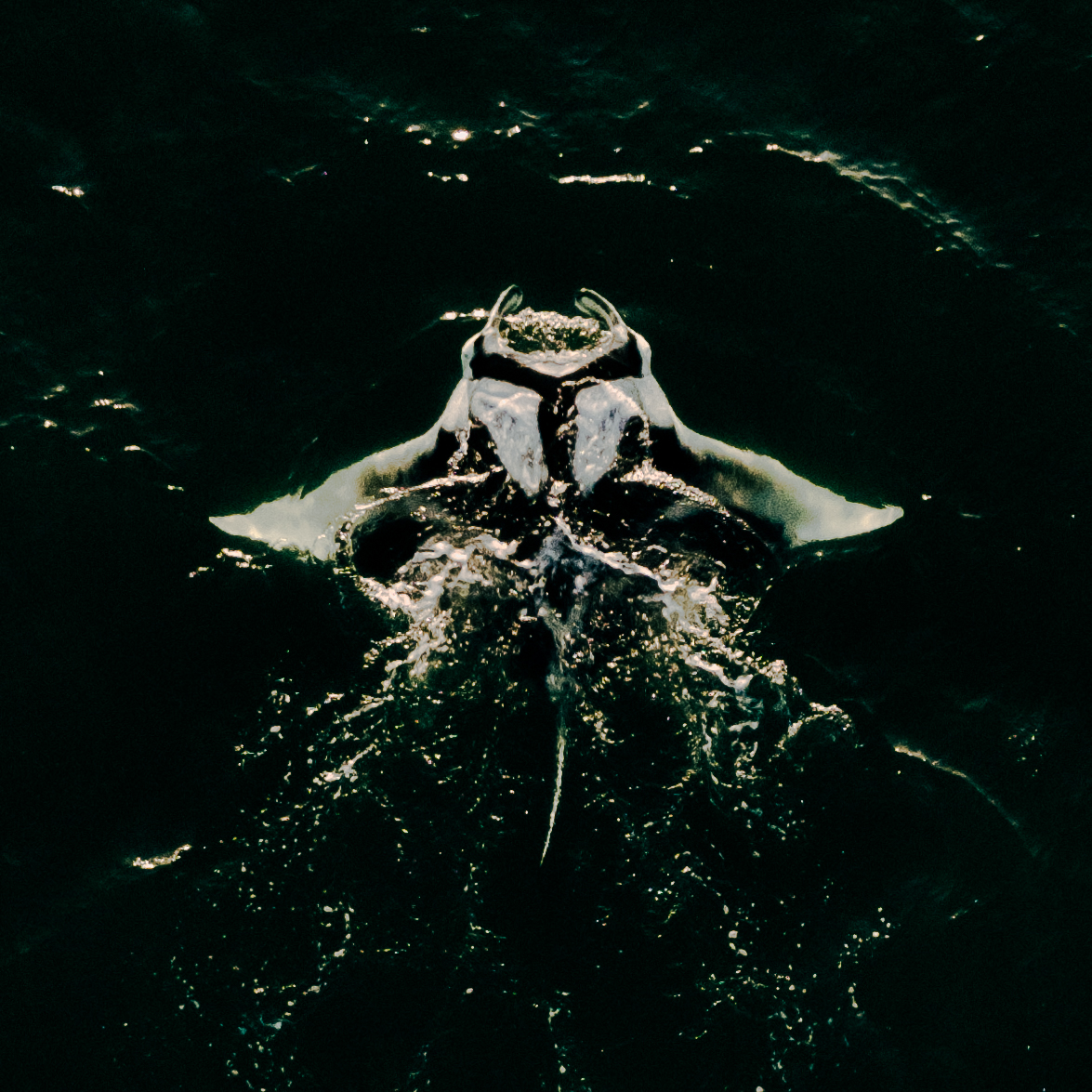

Supplement: Supplemental Information 13 [file peerj-10-13883-s013.jpg]

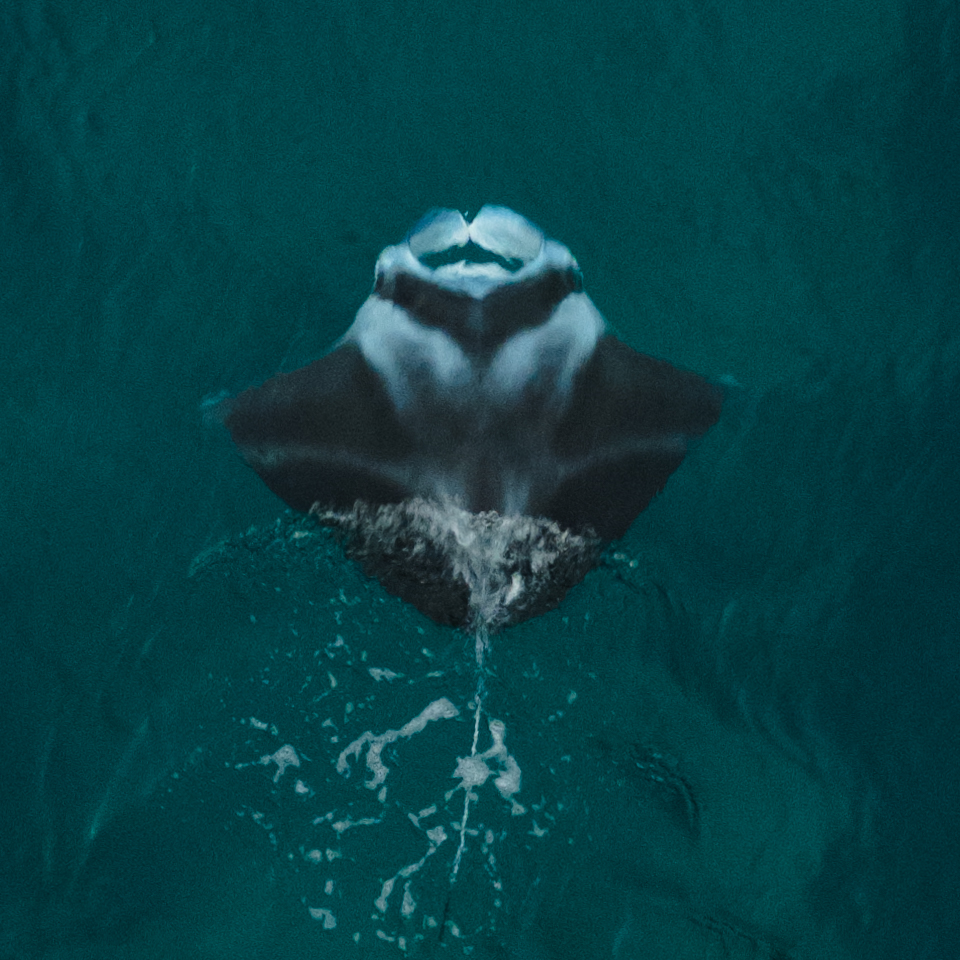

Supplement: Supplemental Information 14 [file peerj-10-13883-s014.jpg]

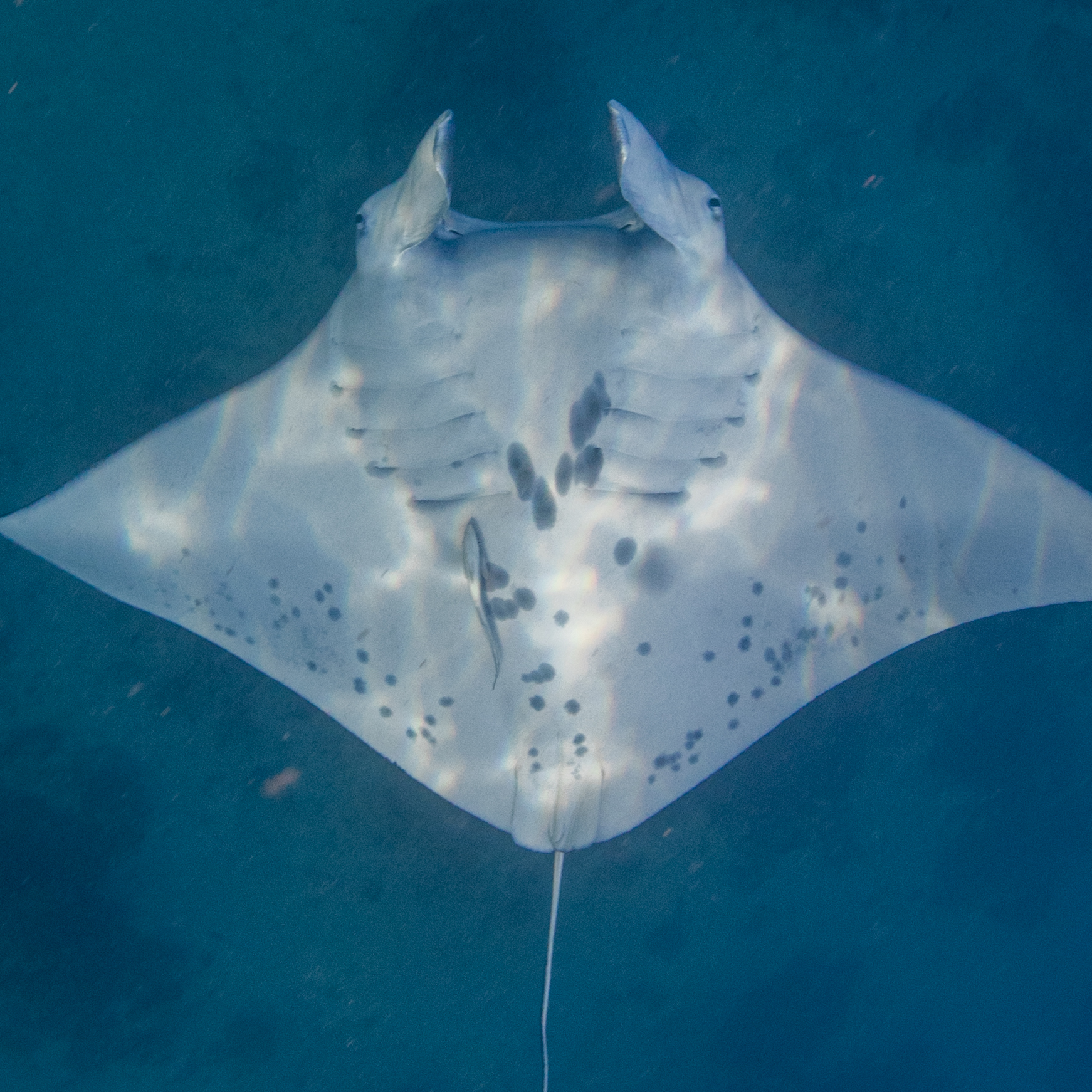

Supplement: Supplemental Information 15 [file peerj-10-13883-s015.jpg]
